# Flexible Inkjet-Printed Heaters Utilizing Graphene-Based Inks

**DOI:** 10.3390/s22031173

**Published:** 2022-02-03

**Authors:** Dimitris Barmpakos, Vassiliki Belessi, Nikolaos Xanthopoulos, Christoforos A. Krontiras, Grigoris Kaltsas

**Affiliations:** 1 microSENSES Laboratory, Department of Electrical and Electronics Engineering, University of West Attica, 122 43 Athens, Greece; gkaltsas@uniwa.gr; 2 Department of Graphic Design and Visual Communication, University of West Attica, 122 43 Athens, Greece; vbelessi@uniwa.gr; 3 Physics Department, University of Patras, 265 04 Patras, Greece; nijoxan@upatras.gr (N.X.); chkron@upatras.gr (C.A.K.)

**Keywords:** flexible heater, printed heater, graphene electronics, functionalized reduced graphene oxide, flexible electronics

## Abstract

Thermal sensors are mainly based on the selective heating of specific areas, which in most cases is a critical feature for both the operation and the performance of the thermal device. In this work, we evaluate the thermoelectrical response of two graphitic materials, namely (a) a commercial 2.4%wt graphene–ethyl cellulose dispersion in cycloxehanone and terpineol (G) and (b) a custom functionalized reduced graphene oxide (*f-*rGO) ink in the range of −40 to 100 °C. Both inks were printed on a flexible polyimide substrate and the Thermal Coefficients of Resistance (TCR) were extracted as TCR_G_ = −1.05 × 10^−3^ °C^−1^ (R^2^ = 0.9938) and TCR*_f-_*_rGO_ = −3.86 × 10^−3^ °C^−1^ (R^2^ = 0.9967). Afterward, the inkjet-printed devices were evaluated as microheaters, in order to exploit their advantage for cost-effective production with minimal material waste. *f-*rGO and G printed heaters reached a maximum temperature of 97.5 °C at 242 mW and 89.9 °C at 314 mW, respectively, applied by a constant current source and monitored by an infrared camera. Repeatability experiments were conducted, highlighting the high robustness in long-term use. The power–temperature behavior was extracted by self-heating experiments to demonstrate the ability of the devices to serve as heaters. Both static and dynamic evaluation were performed in order to study the device behaviors and extract the corresponding parameters. After all the experimental processes, the resistance of the samples was again evaluated and found to differ less than 13% from the initial value. In this work, fabrication via inkjet printing and demonstration of efficient and stable microheaters utilizing a custom ink (*f-*rGO) and a commercial graphene ink are presented. This approach is suitable for fabricating selectively heated geometries on non-planar substrate with high repeatability and endurance in heat cycles.

## 1. Introduction

Selective controllable heating of predefined areas in electronic devices is a key feature for various sensors operation. For example, 2D thermal flow sensors utilize a heating element and exploit variations in the thermal field distribution, which are detected by a set of peripheral sensors to accurately extract the flow vector [1,2]. In chemical sensors, microheaters are usually utilized for either enhancing detection performance or assistance in degassing [3,4]; more specifically, in metal–oxide gas [5], ammonia [6], fully printed SnO_2_ gas [7], HCHO [8], and humidity [9,10] sensors. Microheaters are also quite common in numerous biodevices, where the operating temperature requires precise control [11,12,13,14,15]; analytical paper-based devices incorporating such printed heaters have been developed [13], alongside local thermotherapeutic printed devices [14] and devices for drug metabolism research [15]. Various additive deposition techniques such as printing have been reported for effective selective patterning and fabrication of such devices on flexible [16,17,18,19,20] and stretchable substrates [21]. Liu et al. [17] highlighted the main challenges faced in designing such flexible heaters; material selection, substrate selection, and deposition technique. Additionally, in this comprehensive review, a variety of applications are referenced, such as epidermal thermotherapy, defogging and deicing, and various wearable devices for local temperature monitoring and control. Falco et al. [18] recently studied a handful of properties of CNT-based flexible heaters on polyimide and the findings, amongst others, were the high device stability and repeatability after an initial burn-in process. Wang et al. [20] exhibited a set of thin film heaters based on silver nanowires, on glass and PET substrates, with high spatial control of the temperature distribution, due to the good patterning definition of the devices.

Graphene-based materials are widely used for the development of printed electronics [22,23,24], exhibiting high mechanical durability, resistance to oxidation and corrosion, and good conductivity. Heaters based on graphene or reduced graphene oxide have been shown to reach high temperatures and good mechanical durability and maintain heating capabilities after bending cycles. Yao et al. [25] demonstrated an additively-manufactured graphene-based heater structure with rapid response to temperature changes and precise temperature control. Additional efforts have been presented recently with screen-printed graphene heaters [26]. Typically, graphene heaters can be developed by transferring graphene onto a substrate via heat press [27], dip-coating [28], or evaporation casting [29]. Alternatively, laser-induced graphene has been utilized for selective patterning of such structures [30,31]. Moreover, transparent and flexible microheaters based on CVD-grown graphene have been presented recently [32,33]. Functionalized reduced graphene oxide has also been investigated for heating applications [34,35].

Inkjet printing for electronics exhibits a set of advantages over other additive manufacturing processes [36,37] such as a mask-less digitally-controlled process that offers rapid prototyping capabilities and relatively large scalability and throughput. Additive printing also offers minimal processing byproducts and capabilities to combine materials and structures that would be impossible with traditional subtractive processing [36]. In this work, we evaluated the performance of two graphene-based inks for the development of printed heaters on a flexible polyimide substrate using inkjet technology. This approach allows for cost-effective mass production of custom-patterned heaters with minimal material waste. These materials have been evaluated for their thermal response as temperature sensors [38], and their performance was an indicator of their ability to operate as printed microheaters.

## 2. Materials and Methods

### 2.1. Materials

Graphite (powder, synthetic, particle size <20 μm), potassium chlorate (purum >99.0%) and 2,4-diaminobenzenesulfonic acid (≥98%) were purchased from Merck, KGaA, (Darmstadt, Germany). The solvents nitric acid (65%) and sulfuric acid (95–97%) were purchased from Riedel-de Haen (Munich, Germany) and Merck, KGaA, (Darmstadt, Germany), respectively, and were used as provided. A commercial graphene ink (code 793663) with 2.4 wt% solids (graphene and ethyl cellulose) in cyclohexanone and terpineol was used for inkjet printing, which according to the datasheet requires a minimum curing temperature of 250 °C.

### 2.2. Preparation and Characterization of f-rGO

An aqueous graphene oxide (GO) dispersion (1 mg/mL) was prepared following a previously described method [38,39]. Then, an appropriate amount of 2,4-diaminobenzenesulfonic acid (2,4-DBSA) was added in the GO dispersion using a mass ratio GO:2,4-DBSA equal to 1:3. The mixture was refluxed under magnetic stirring for 2 h and, after cooling, was vacuum filtered using suitable Nylon membrane filters (0.45 μm pore size, Whatman). The product was washed extensively with deionized water, ethanol, and acetone and air-dried by spreading on a glass plate. Then, an appropriate amount of the as prepared *f-*rGO powder was promptly dispersed in water using an ultrasound bath cleaner (Branson 3800, 110 W, 40 kHz), and the ink was prepared (~3.6 mg *f*-rGO/g). The morphology of the *f-*rGO and G was evaluated using Scanning Electron Microscopy (SEM) (JEOL JSM-6510LV).

### 2.3. Formation of Printed Heaters and Measurement Setup

**Figure 1:**
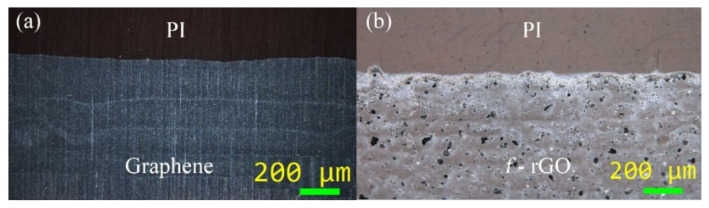
Printed structures on Kapton substrate: graphene (**a**) and *f-*rGO (**b**).

Droplet generation analysis and inkjet settings have been covered in a previously work [38]; in short, graphene and *f-*rGO inks were inkjet-printed using unipolar square wave pulses of 90 V amplitude for 10 μs and −90 V for 50 μs, respectively, while droplet spacing was set at 65 μm for both axes. A Thetametrisis FR-DEPOSIT (Athens, Greece) drop-on-demand piezoelectric inkjet printer was used for the sample formation. Polyimide substrate (125 μm-thick, DuPont Kapton HN, Wilmington, Delaware, USA) was treated with 1 M NaOH for 7 min to increase wettability. Prior to printing, the substrate was rinsed with acetone, deionized water, and isopropyl alcohol. Lines of 5 cm × 500 μm were printed on Kapton, and the samples were cured appropriately in an air convection oven, at 240 °C for 1 h. Top view optical microscopy images of both printed inks are illustrated in Figure 1.

The thermal–electrical characterization was performed in a modified Novocontrol BDS 1200 sample cell in order to facilitate the planar electrical configuration of the samples. Current–voltage measurements were performed through a Keithley 2611 A sourcemeter in the temperature range between −40 and 100 °C in steps of 20 °C and voltage range from −5.0 V to +5.0 V. The temperature of the samples was controlled by the Quatro temperature control system provided by Novocontrol with a temperature stabilization of ±0.2 °C. Current–voltage measurements were repeated in several successive days to confirm the repeatability of the electrical response of the specimens.

For the evaluation of the heaters, an additional setup was used consisting of a probe-station connected with a Keithley 2612 SourceMeter, which was utilized for both supplying constant current and measuring voltage. A FLIR A655SC IR camera was used to monitor the samples temperature.

## 3. Results and Discussion

**Figure 2:**
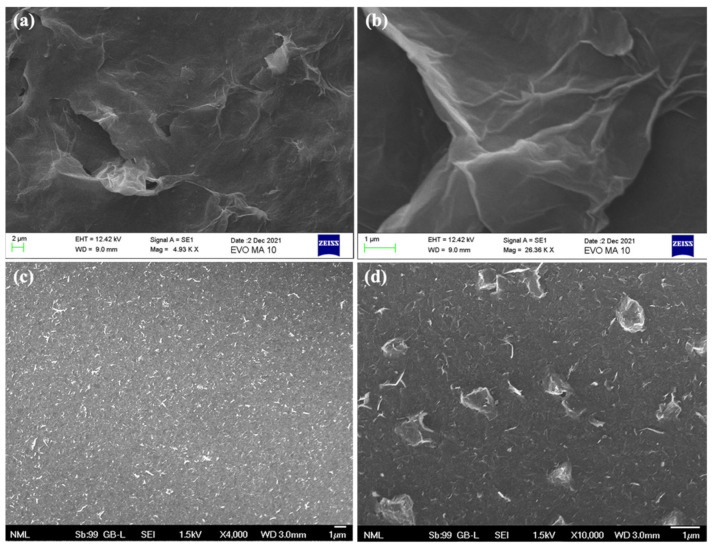
SEM images of *f-*rGO (**a**,**b**) and SEM images of graphene (**c**,**d**).

The as-prepared conductive *f-*rGO is a highly dispersible and stable material in water and other solvents [37] with excellent shelf life. It is prepared via a reliable, affordable, facile, one-pot method that allows the simultaneous reduction and functionalization of GO using sulfonated aromatic diamines. Furthermore, our previous studies have shown that due to these properties, it is a promising material in the field of printed electronics [38,39,40], for example, as a pigment in conductive water-based inks for gravure and flexography or as an ink for inkjet-printed temperature sensors. In this work, a water dispersion of this highly hydrophilic material was used without any surfactant or additive. On the other hand, the commercial graphene ink is based on a mixture of solvents (cyclohexanone and terpineol), while ethyl cellulose is used as the stabilizing polymer [41]. In Figure 2 are presented representative SEM images of the *f-*rGO wrinkled nanosheets and the commercial graphene dispersion.

**Figure 3:**
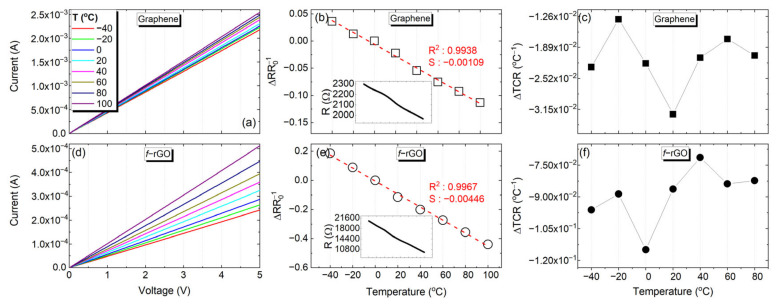
IV curves, relative resistance change with respect to temperature and Thermal Coefficient of Resistance dependency on temperature in the range of −40 to 100 °C for both graphene and *f-*rGO.

For extracting the I-V curves and the corresponding electrical resistance of the specimens, the cryostat system was used, as described in the previous section. Figure 3a,d present the I-V curves with respect to the temperature for graphene and *f*-rGO, respectively. Each line was extracted after the cryostat system set and stabilization at the corresponding temperature. The slope of each curve indicates the factor 1/R, where R is the resistance of the specimen. From the abovementioned figures, it can be extracted that, in accordance with previous findings [35], the graphene–ethyl cellulose dispersion in cyclohexanone–terpineol presented higher electrical conductivity than the aqueous *f-*rGO dispersion by approximately one order of magnitude. A remarkable linearity in the I-V curves was observed throughout the entire evaluated temperature range for both inks, which is indicating in Figure 3b,e, where the relative resistance change (ΔR/Ro) is plotted as a function of temperature for graphene and *f*-rGO, respectively (Ro is the resistance of each material at 0 °C). In the inset of Figure 3b,e, the absolute value of the resistance is indicated as a function of temperature for both materials. The abovementioned remark is verified by the corresponding mean and standard deviation of the IV curves in all the evaluated temperature values, which were extracted as R^2^_G_ = 0.9998 ± 2.78 × 10^−6^ and R^2^*_f-_*_rGO_ = 1.0000 ± 7.15 × 10^−7^. The same curve was mirrored when applying negative voltages of same amplitude, highlighting a completely ohmic behavior throughout the investigated range. The Mean Thermal Coefficient of Resistance (TCR) was extracted in the range of −40 to 100 °C. The corresponding values were TCR_G_= −1.09 × 10^−3^ °C^−1^ with R^2^ = 0.9938 for graphene and TCR*_f-_*_rGO_ = −4.46 × 10^−3^ °C^−1^ with R^2^ = 0.9967 for *f-*rGO. Figure 3c,f present the difference in the extracted TCR value as a function of the evaluated temperature for both materials. It can be easily observed that the variations are an order of magnitude lower than the actual TCR values, indicating that the TCR can be considered stable within the examined temperature range. Moreover, this behavior shows the capabilities of both materials to act as temperature sensors and as heaters with a well-defined response.

**Figure 4:**
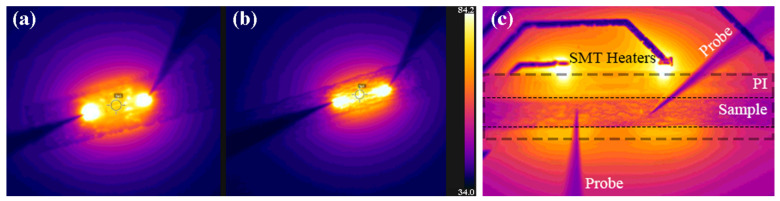
Graphene under 8 mA (**a**), *f-*rGO under 4 mA (**b**), and a sample with the temperature stabilization SMT heaters underneath (**c**).

For assessing the functionality of both materials as printed microheaters, a set of experiments was carried out. Initially, both structures were supplied with a constant current for reaching a target maximum temperature of approximately 90 °C. Graphene heaters were supplied with six successive current pulses of 1.33, 2.67, 4.00, 5.33, 6.67, and 8.00 mA, resulting in a maximum temperature of 90.3 °C. *f-*rGO heaters, similarly, were supplied with six successive current pulses of 0.66, 1.33, 2.00, 2.67, 3.33, and 4.00 mA, resulting in a maximum temperature of 97.5 °C. The substrates were maintained in a stable temperature environment of 35 °C (room temperature) by a custom PCB circuit with standard Pt100 heater elements, which were driven with a constant current (Figure 4c). With the specific approach, the Pt100 elements can be used as heaters and temperature sensors; thus, the substrate temperature was continuously monitored both by the IR camera and the Pt100 elements simultaneously. The experimental evaluation revealed that both materials can be effectively used as printed heaters with relatively high-power efficiency. *f-*rGO can reach 100 °C at 250 mW, while graphene was able to elevate at the same temperature with 320 mW input power (28% more power) progressively from room temperature. Indicative IR pictures of the experimental evaluation of printed heaters experimental evaluation are presented in Figure 4a,b.

**Figure 5:**
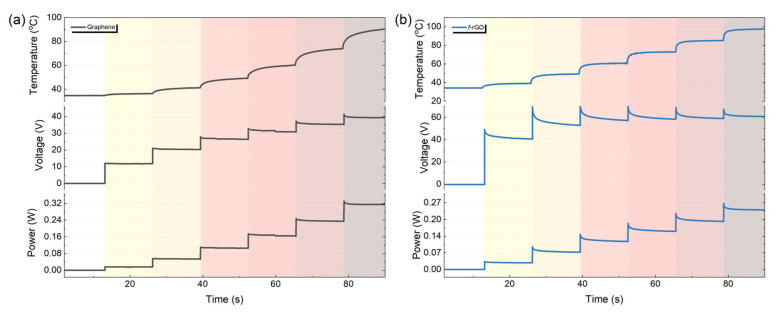
Graphene under 0–8 mA pulses (**a**), *f-*rGO under 0–4 mA (**b**).

Figure 5 presents the electrical response of the heaters with respect to temperature. In any case the temperature increases with increasing current pulses and reaches a plateau within a few seconds, indicating that the proposed microheaters can respond relatively fast to different set values of temperature. The corresponding rise time is directly affected by the thermal properties of the substrate. An overshoot appears in the beginning of each voltage step, and the effect is more obvious in the *f-*rGO case. This is caused by the self-heating of the material when a current pulse is applied. The pulse induces a sudden input power increase, which leads to a temperature increase. Both materials exhibit negative TCR values, indicating that the increase in temperature will result in a decrease in the corresponding resistance value, which induces a secondary power reduction effect, as the applied current is constant (P = I^2^R). This phenomenon is actually negative feedback to the temperature of the samples, and it is demonstrated by the observed spikes at the beginning of each pulse. It is more obvious in the *f-*rGO case because this material exhibits a higher absolute value of TCR.

**Figure 6:**
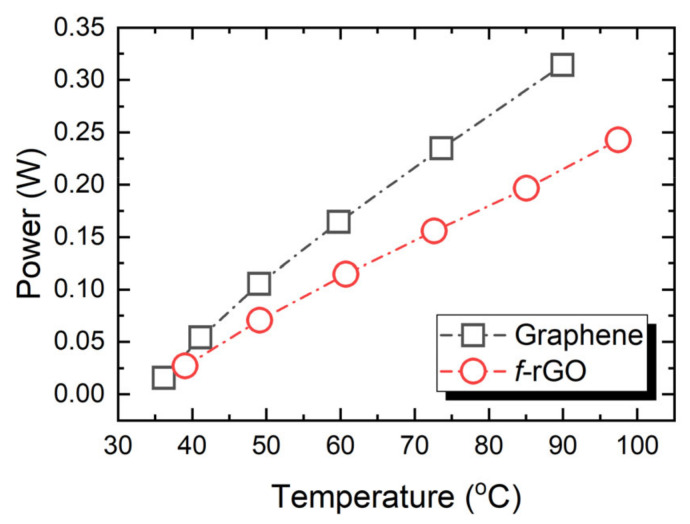
The temperature increase as a function of applied power for Graphene and *f-*rGO heaters.

The power–temperature relationship for both materials is presented in Figure 6. The *f-*rGO implements a more efficient heater device, since it can reach higher temperatures with the same applied power on the same substrate.

**Figure 7:**
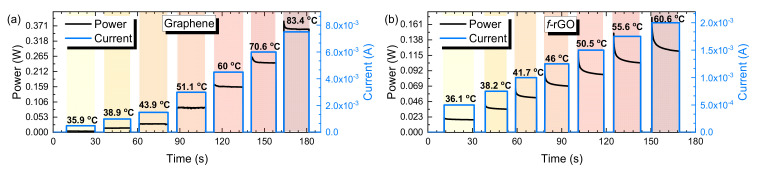
Graphene sample with a current sweep of 0–7.5 mA (**a**) and *f-*rGO sample with a current sweep of 0–2 mA (**b**). Following each pulse, the current supply was shut down, and the sample was able to reach room temperature.

Performance of the printed heaters’ response to incremental return-to-zero pulses was also evaluated as illustrated in Figure 7. For each sample, a current sweep was performed, with each current pulse being followed by current supply cut-out. That way, the capability of the system to reach steady state from a room temperature was monitored. A power overshooting in the beginning of each current step is also observed here, which is mainly due to the materials’ negative TCR. With increasing temperature, the resistance drops; therefore, for an application of a constant current this behavior leads to a decrease in power until a steady-state temperature was reached. This effect is important and should be taken into consideration in the designing of microheaters, since it affects the actual steady state of the device; furthermore, it also induces a temporary temperature overshoot that it might damage the device.

**Figure 8:**
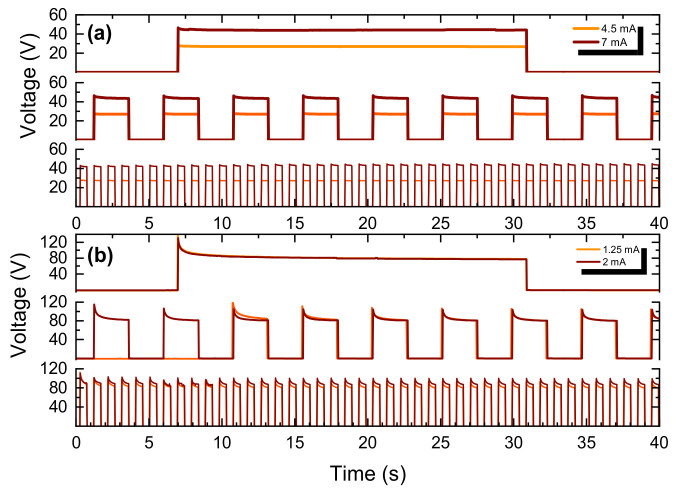
Reliability–stability test for both materials for different currents and different pulse durations; graphene (**a**) and *f-*rGO (**b**).

Reliability evaluation was performed by heating cycles of different intervals, for assessing the samples’ ability to withstand consecutive heating and cooling (Figure 8). For each sample, two current amplitudes and three pulse sequences of different intervals [0.5 s (900 pulses), 2.5 s (360 pulses), and 25 s (36 pulses)] were applied for a period of approximately 15 min. For all pulse durations and currents, the response was similar throughout the experiment, and neither the sample nor the pulse morphology changed.

**Figure 9:**
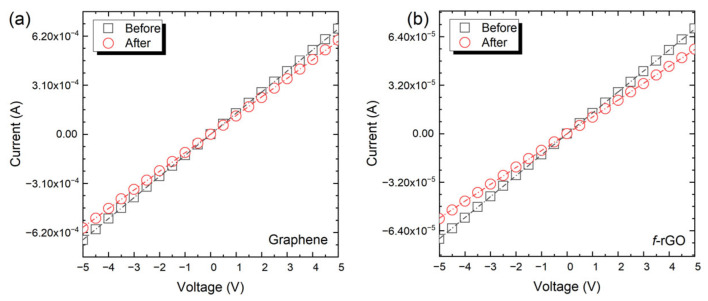
I-V curves before and after the thermal evaluation for Graphene (**a**) and *f-*rGO (**b**).

Finally, to assess the durability of the devices, the same I-V curves were extracted after all the aforementioned experiments (Figure 9). The total resistance change was found to be in the range of 10%; similar thermal experiments were repeated, but no additional resistance change was observed, highlighting the durability to thermal stress and its ability to maintain a stable electrical response.

## 4. Conclusions

In this work, a complete thermal and electrical response of two inkjet-printed materials was presented with a focus on printed heater applications. The Thermal Coefficient of Resistance was extracted as TCR_G_ = −1.05×10^−3^ °C^−1^ and TCR*_f-_*_rGO_ = −3.86×10^−3^ °C^−1^ in an extensive range of temperatures (−40 to 100 °C) and highlighted the superiority of *f-*rGO behavior, in accordance with previous findings. Furthermore, the simple *f-*rGO fabrication process and its cost (compared to graphene) may further enhance its implementation in thermal device applications. Inkjet structures of both materials were thermally evaluated in order to demonstrate their ability to act as heaters in printed devices. *f-*rGO appears to be a more efficient approach since it can reach a maximum temperature of 100 °C with 250 mW, while graphene requires 28% more power (320 mW) to reach the same temperature on a 125 μm-thick Kapton substrate. The power–temperature relationship for both materials was extracted, indicating the ability of both devices to act as heating elements. The dynamic response of the printed heaters showed that an overshoot appears at the beginning of each voltage step, which is caused by the self-heating of the material as a result of sudden power increase in combination with the negative TCR values of both materials. The effect is more obvious on the *f-*rGO case and should be taken into consideration in the estimation of the steady state temperature value of the printed structures. Reliability evaluation was also performed by a series of successive heating cycles of different intervals, which proved the stable response of the printed heaters and their durability to thermal stressing.

Future steps include integration of the proposed materials in a sensor device for utilizing their heating capabilities, where sensitivity can be increased with elevated temperature and degassing can be significantly enhanced. Furthermore, different experiments have already been programmed, with different material concentration and printing passes, to assess the importance of these parameters in device operation.

## Data Availability

The data presented in this study are available on request from the corresponding author.
